# Novel Method for Characterizing Humic Substances Using Fluorescent Solvatochromism

**DOI:** 10.3390/s26010107

**Published:** 2025-12-23

**Authors:** Kazuto Sazawa, Hanae Koyama, Yusuke Yamazaki, Yoshiki Hara, Nozomi Kohama, Yustiawati Yustiawati, Hideki Kuramitz

**Affiliations:** 1 Department of Natural and Environmental Sciences, Faculty of Science, University of Toyama, 3190 Gofuku, Toyama 930-8555, Japan; 2 Hydrogen Isotope Science Research Center, University of Toyama, 3190 Gofuku, Toyama 930-8555, Japan; 3 Research Center for Limnology and Water Resources, National Research and Innovation Agency, Jl. Raya Jakarta-Bogor Km. 46, Cibinong, Bogor 16911, Indonesia

**Keywords:** fluorescent probes, humic substances, polarity, solvatochromism

## Abstract

Charge-transfer-type fluorochromes, which exhibit shifts in fluorescence intensity and emission wavelength in response to solvent polarity changes, have been widely employed to investigate solute–solvent interactions. Humic substances (HSs) are naturally occurring macromolecular organic acids derived from plant residues, with structural properties that vary depending on their origin and environmental conditions. The polarity of HSs is closely associated with the mobility and toxicity of environmental pollutants, making their chemical characterization essential. In this study, we developed a rapid and straightforward method to characterize HS polarity using fluorescent solvatochromism. The fluorescence peak shifts of four dyes—8-anilino-1-naphthalenesulfonic acid (ANS), acridine orange (AO), methylene blue (MB), and Rhodamine B (RhB)—were evaluated in the presence of humic acids (HAs), a major component of HSs. To assess environmental variability, a total of twelve HS samples were tested, including HSs derived from soils of different origins, compost, commercial reagents, and standard reference materials. Among these, AO and MB exhibited distinct spectral shifts without overlapping with the intrinsic fluorescence of HAs. Notably, MB displayed a consistent blue shift dependent on HA concentration, with the most stable response observed at 5 mg/L. The magnitude of this shift was significantly correlated with UV–Vis parameters associated with the aromaticity, humification degree, and polarity of HSs. Overall, this study demonstrates that MB-based fluorescent solvatochromism can function as an empirical and facile indicator for assessing the structural and microenvironmental characteristics of HSs, providing a rapid and complementary screening approach for HSs extracted and purified from environmental samples.

## 1. Introduction

Colorimetric and fluorescence-based detection strategies have emerged as powerful analytical approaches for evaluating complex environmental samples due to their high sensitivity, operational simplicity, and adaptability to diverse chemical environments. Chromism refers to a reversible color change triggered by external stimuli such as light, temperature, solvent polarity, or pH variation [1,2,3]. Among its various forms, fluorescence solvatochromism is a phenomenon wherein the fluorescence wavelength of a molecule shifts in response to changes in the surrounding polarity, even in dilute solutions where interactions between dye molecules are negligible [3,4,5,6]. Fluorescent solvatochromic dyes are widely used to assess intracellular physical microenvironments. Classical dyes such as 6-dodecanoyl-2-(dimethylaminonaphthalene) (LAURDAN) and 6-propionyl-2-dimethylaminonaphthalene (PRODAN) have long been used to probe membrane-associated polarity and lipid packing, while recent studies report advanced probes with red-shifted emission and improved performance [4,5]. These developments have further enabled the visualization of lipid droplets, which represent low-polarity intracellular domains closely linked to cellular metabolism and disease diagnostics [6].

Humic substances (HSs) are naturally occurring, ubiquitous high-molecular-weight organic acids widely distributed in soil alongside rivers, lakes, and other freshwater environments [7]. Among these substances, humic acids (HAs) constitute a major fraction. Both HAs and fulvic acids (FAs) are soluble at pH values above two. Moreover, HAs exhibit a range of properties—including persistence, redox capacity, surface activity, complexation with metal ions, and both hydrophobic and hydrophilic characteristics—that are shaped by their environmental formation conditions and origin. These properties, in turn, influence the transport of nutrients and the toxicity of hydrophobic organic pollutants (HOPs) and metals [7,8,9]. Accordingly, evaluating the chemical characteristics of HSs is essential. However, the structural complexity and diversity of these acids necessitate the use of multiple characterization techniques, including functional group analysis, elemental analysis, ultraviolet–visible (UV-Vis) spectroscopy, fluorescence spectroscopy, and size exclusion chromatography [10,11,12,13,14,15]. In recent years, advanced analytical techniques such as Fourier transform ion cyclotron resonance mass spectrometry (FT-ICR-MS), spectroscopic and electrochemical methods, and complementary LC–MS/GC–MS analyses have been employed to provide more detailed molecular-level insights into the structure and composition of humic acids [16,17,18]. In parallel, advanced spectroscopic approaches—particularly excitation–emission matrix (EEM) fluorescence spectroscopy combined with parallel factor analysis (PARAFAC) and two-dimensional correlation spectroscopy (2D-COS)—have been widely applied to elucidate the interaction mechanisms between HSs and environmental contaminants. These methods enable detailed analysis of the binding behavior and interaction characteristics of HSs with hydrophobic organic pollutants and metal ions [19,20,21,22]. Although recent advances have enabled increasingly detailed analysis of HAs, many of these techniques are complex and labor-intensive. Therefore, the development of simple and rapid evaluation methods for HSs remains an important and complementary research direction.

In this study, we hypothesized that fluorescent solvatochromism—reflecting the chemical properties of HSs, particularly their polarity—can be detected through the appropriate selection of fluorescent dyes and optimization of measurement conditions. Because the structural characteristics of HSs—such as aromaticity, functional group composition, polarity, and molecular size—are closely associated with the mobility and toxicity of environmental pollutants [7,8,9], establishing a simple and reliable method for their evaluation is of great importance. Accordingly, this study aimed to develop a new characterization method for HSs using fluorescent solvatochromism. To our knowledge, this is the first study to apply solvatochromic dyes for direct polarity-based characterization of HAs. To this end, we employed four fluorescent dyes—8-anilino-1-naphthalenesulfonic acid (ANS), known for its solvatochromic properties [23,24,25]; acridine orange (AO) [26,27]; methylene blue (MB) [28]; and rhodamine B (RhB) [29]—as fluorescent probes. We first examined shifts in their emission wavelengths in solvents of varying polarity, including tetrahydrofuran (THF), acetone (ACTN), dimethyl sulfoxide (DMSO), and ethanol (EtOH). To evaluate their responses to natural materials, five types of HAs extracted from soils of different origins (peatlands, compost, and forest soils), two commercial reagents, and five standard samples (including both HAs and FAs) were tested. By comparing the emission peak positions of the dyes in the presence of HAs, we determined the optimal dye and HA concentrations for evaluation based on the observed peak shifts and fluorescence quenching. Additionally, we assessed the relationships between the peak shift magnitudes and various chemical parameters of HSs, including aromaticity, degree of humification, functional group content, molecular weight and polarity based on elemental ratios. This approach offers a rapid optical alternative to conventional HA analyses and establishes a new polarity-based fluorescence method for characterizing HSs.

## 2. Materials and Methods

### 2.1. Chemicals

ANS was purchased from Tokyo Chemical Industry Co., Ltd. (Tokyo, Japan). ACTN, AO, MB, RhB, THF, DMSO, and EtOH were obtained from Fujifilm Wako Pure Chemical Industries, Ltd. (Osaka, Japan). A 0.1 M phosphate buffer (PB, pH 7.0) was prepared using NaH_2_PO_4_ and Na_2_HPO_4_. All reagents were of analytical grade, and sterile deionized water was used throughout. Stock solutions of each dye (ANS, AO, MB, and RhB) were prepared at 200 µM. Working solutions were then diluted to 10 µM in various solvents. Five types of HAs previously extracted and purified in our earlier studies from soils of different origins—including peatlands (*Amou* peat humic acid: APHA, *Kalimantan* peat humic acid: KPHA, and *Shinshinotsu* peat humic acid: SPHA), forest soils (Forest soil humic acid: FSHA), and compost (Compost humic acid: CHA)—were employed in this study [8,14,15]. The extraction and purification of each HA sample were conducted according to the International Humic Substances Society (IHSS) standard method. In addition, two commercially obtained humic acids, Aldrich humic acid (AHA, Sigma-Aldrich Co. LLC., St. Louis, MA, USA) and Wako humic acid (WHA, Fujifilm Wako Pure Chemical Industries, Ltd., Osaka, Japan), were also used, and both samples were purified prior to use by alkaline dissolution followed by HF/HCl treatment, dialysis, and freeze-drying. Five distinct standard HA and FA reference materials were obtained from the IHSS, namely Elliott Soil IV HA (ESHA), Pahokee Peat I HA (PPHA), Leonardite HA (LHA), Suwannee River I FA (SRFA), and Pahokee Peat II FA (PPFA). Prior to experimentation, purified HA and FA were dissolved in 1 M NaOH (pH > 10) and stirred for 30 min [8,14,15]. The pH was then adjusted to 8.0 using 1 M HCl. A stock solution was prepared at 100 mg/L. Artificial seawater was prepared using the following constituents: H_3_BO_3_ (0.5 mM), KBr (0.85 mM), NaHCO_3_ (2.79 mM), KCl (9.3 mM), CaCl_2_·2H_2_O (10.23 mM), Na_2_SO_4_ (28.8 mM), MgCl_2_·6H_2_O (54.6 mM), and NaCl (420 mM) [30].

### 2.2. Characterization of HAs

The UV-Vis parameters (*A*_400_/*A*_600_, *E*280, *E*2/*E*3 (the absorbance ratio at 250/365 nm)), functional group content, elemental composition, molecular weight, log *K*_OM_ of Ant, and log *K*_ML_ of Hg for the HAs (APHA, CHA, FSHA, KPHA, SPHA, AHA and WHA) were obtained from our previously reported analytical results. *A*_400_/*A*_600_ is known to correlate with the *E*4/*E*6 parameter (the absorbance ratio at 465/665 nm), which is an established index of the degree of humification [13]. These values are summarized in Table S1 [8,14,15]. The UV-Vis parameters and molecular weights of the IHSS standard HAs and FAs were determined using established methods (Supplementary Materials). Acidic functional groups and elemental compositions were referenced from the IHSS website (https://humic-substances.org/). In addition, the polarity index (PI) was calculated based on the elemental composition data provided by IHSS (Table S2).

### 2.3. Fluorescence Analysis of the Dyes and HAs

The three-dimensional excitation–emission matrix (3DEEM) fluorescence spectral profiles of the dyes and HAs (APHA, CHA, FSHA, KPHA, SPHA, AHA and WHA) were recorded using a fluorescence spectrophotometer (RF-6000; Shimadzu, Kyoto, Japan). The measurements were performed at room temperature. The scanned wavelength ranges were Ex./Em. = 200–600 nm/300–600 nm for HAs and Ex./Em. = 300–700 nm/300–700 nm for dyes. The scan speed was 2000 nm/min, and both the excitation and emission slit widths were set to 5 nm. The relative fluorescence intensity was calibrated in quinine sulfate units (QSU), where 1 QSU corresponds to 1 μg/L of quinine sulfate monohydrate in 0.05 M H_2_SO_4_ at Ex./Em. = 355/450. Milli-Q water (Millipore Co. Ltd., Darmstadt, Germany) was used as a blank in all measurements.

### 2.4. Fluorescence Spectral Analysis of Dyes in the Presence of HA

In a 20 mL volumetric flask, 2 mL of HA solution and 1 mL of 200 µM dye solution were mixed. The mixture was diluted to 20 mL with PB (pH 7), yielding a final dye concentration of 10 µM. The fluorescence spectra were then recorded using the RF-6000 spectrophotometer (Shimadzu Co., Kyoto, Japan). The fluorescence spectra of MB in the presence of each HS were measured at 0.1 nm wavelength intervals.

### 2.5. Data Analysis

The spectral peaks of MB in the presence and absence of HA were analyzed using Origin software (2022 SR1) by applying the two-point baseline method combined with the Savitzky–Golay smoothing filter and first-derivative processing. Savitzky–Golay smoothing was performed with a window size of 31. Linear regression and Spearman correlation analyses were performed using JMP Pro version 18.0.0 (SAS Institute, Inc., Cary, NC, USA). Heatmaps of Spearman’s correlation was created using the “corrplot” and “ggplot2” packages in R (version 4.0.4; R Core Team, 2021, http://www.r-project.org). The Deming regression was performed using the Deming function in the “MethComp” package in R. Inverse regression analyses were conducted in R using the lm(x~y) model.

## 3. Results and Discussion

### 3.1. Fluorescence Characteristics and Peak Shifts of the Dyes in Various Solvents

**Figure 1:**
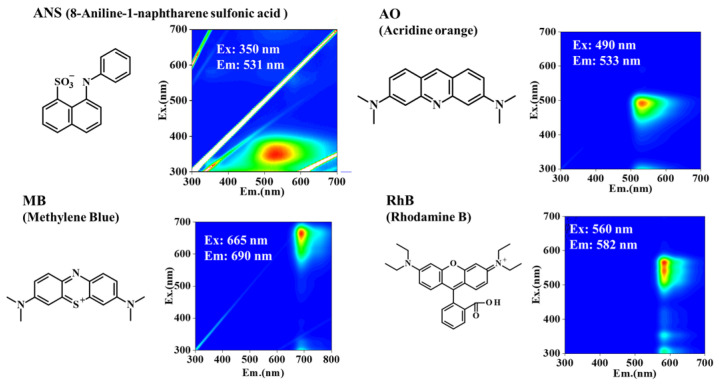
Chemical structures and corresponding three-dimensional excitation–emission matrix (3DEEM) fluorescence spectra of the fluorescent dyes used in this study: 8-anilino-1-naphthalenesulfonic acid (ANS), acridine orange (AO), methylene blue (MB), and rhodamine B (RhB). In the 3DEEM plots, the *x*-axis represents the emission wavelength (Em., nm) and the *y*-axis represents the excitation wavelength (Ex., nm).

Figure 1 presents the three-dimensional excitation–emission matrix (3DEEM) fluorescence spectra of the fluorescent dyes at a concentration of 10 μM. Notably, fluorescence peaks were observed at Ex/Em = 350 nm/531 nm for ANS, 490 nm/533 nm for AO, 665 nm/690 nm for MB, and 560 nm/582 nm for RhB.

**Figure 2:**
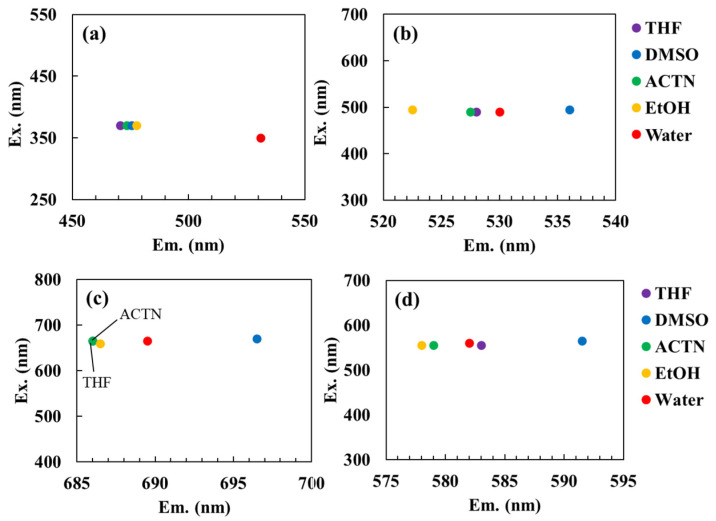
Fluorescence peak shifts of each dye in solvents of varying polarity: (**a**) ANS, (**b**) AO, (**c**) MB, and (**d**) RhB. Solvents include tetrahydrofuran (THF), dimethyl sulfoxide (DMSO), acetone (ACTN), ethanol (EtOH), and water. In the peak position plots, the *x*-axis represents the emission wavelength (Em., nm) and the *y*-axis represents the excitation wavelength (Ex., nm).

Figure 2 displays the fluorescence peak shifts of each dye in solvents of varying polarity, including THF, ACTN, DMSO, EtOH, and water. In all cases, distinct solvent-dependent peak shifts were observed. Notably, the fluorescence quantum yield of ANS in water has been reported to be 0.003 ± 0.001, whereas it is known to increase markedly to 0.71 ± 0.07 in DMSO [25]. This enhancement is attributed to the quenching effect of water molecules on ANS fluorescence [25]. In hydrophilic solvents such as DMSO, fluorescence enhancement occurs because these solvents displace water molecules from the vicinity of excited ANS molecules, thereby reducing quenching by water. Figure S1 illustrates the fluorescence spectra of each dye in different solvents. Relative to water, the fluorescence intensity of ANS increased approximately 140–350 fold in THF, ACTN, DMSO, and EtOH. The maximum emission wavelength (λ_em_) of ANS was 531 nm in water and 475.5 nm in DMSO, consistent with previous reports [25].

Table S3 presents the dielectric constant, molecular dipole moment, refractive index, and *E*_T_(30) value of each solvent [2]. *E*_T_(30) is an empirical solvent polarity scale derived from the pronounced negative solvatochromism of a standard betaine dye, and is widely used to characterize solvent polarity in various solvents and solvent mixtures [31]. The maximum λ_em_ of ANS was highest in water and decreased progressively in EtOH, DMSO, ACTN, and THF, indicating a clear blue shift in solvents with lower *E*_T_(30) values (Figure 2a). Excluding water, a strong correlation was observed between the *λ_em_* value of ANS and the *E*_T_(30) values of the solvents (*r* = 0.978, *p* < 0.05). For AO, MB, and RhB, the λ_em_ values in the tested solvents were ranked as follows: DMSO > water > THF > ACTN > EtOH for AO (Figure 2b); DMSO > water > EtOH > THF = ACTN for MB (Figure 2c); and DMSO > THF > water > ACTN > EtOH for RhB (Figure 2d). Although these solvent-dependent shifts were less distinct than those observed for ANS, AO and MB also exhibited noticeable blue shifts in low-polarity solvents, such as THF and ACTN.

### 3.2. Fluorescence Properties of the HAs Used in This Study

**Figure 3:**
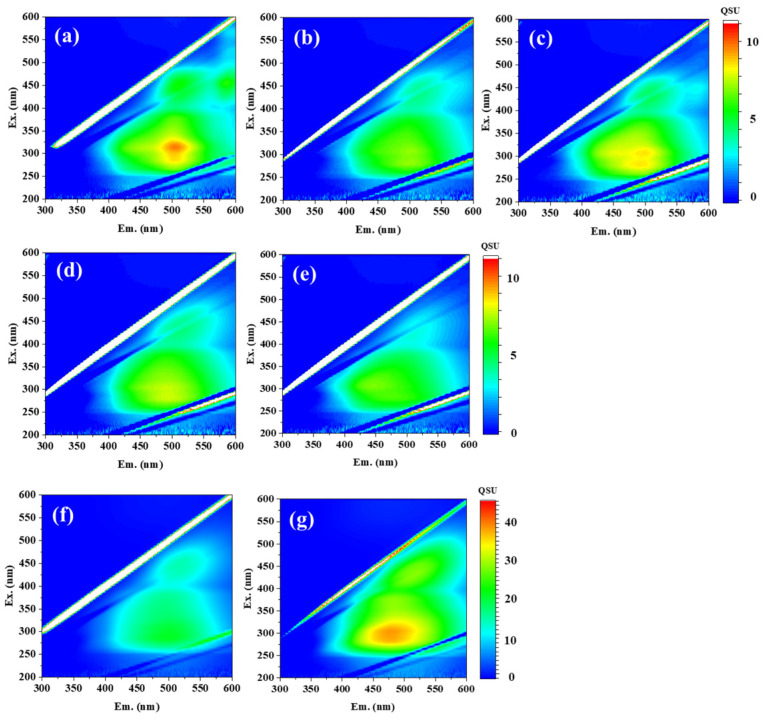
Three-dimensional EEM fluorescence spectra of 10 mg/L humic acids (HAs) extracted from five soil types ((**a**): APHA, (**b**): CHA, (**c**): FSHA, (**d**): KPHA, (**e**): SPHA) and two commercial sources ((**f**): AHA, (**g**): WHA). The bar charts adjacent to each spectrum indicate the contour intervals. In the 3DEEM plots, the *x*-axis represents the emission wavelength (Em., nm) and the *y*-axis represents the excitation wavelength (Ex., nm).

The HAs examined in this study included two commercial reagents (AHA and WHA) and five samples extracted from natural sources: peat (APHA, KPHA, and SPHA), forest soil (FSHA), and compost (CHA). The chemical characteristics of these HAs—including humification degree, aromaticity, functional group content, molecular weight, elemental ratios, and structural type—had been previously analyzed and are summarized in Table S1 [8,14,15]. Figure 3 presents the 3DEEM fluorescence spectra of each HA sample at a concentration of 10 mg/L in PB (pH 7). All samples exhibited two prominent fluorescence peaks: Peak C_1_ (Ex/Em = 280–315/479–502 nm) and Peak C_2_ (Ex/Em = 425–450/513–521 nm), both of which are commonly observed in HAs [9]. In addition, the IHSS website provides the 3DEEM fluorescence spectra for PPHA (1S103H), LHA (1S104H), and PPFA (2S103F), showing that the HAs exhibit peaks around Ex 260 nm/Em 460 nm and Ex 450 nm/Em 500 nm, while the FA shows peaks at Ex 320 nm/Em 420 nm and around Ex 220 nm/Em 420 nm (https://humic-substances.org/ftir-13c-nmr-and-fluorescence-spectra/ (accessed on 1 December 2025)).

Fluorescence indices are widely recognized as effective indicators for assessing the sources and properties of fluorescent components. In particular, the fluorescence index (FI) and biological index (BIX) provide insight into the origins of fluorescent matter, while the humification index (HIX) shows a positive correlation with the degree of humification [32,33]. Table 1 summarizes the positions and intensities of Peak C_1_ obtained from the 3DEEM fluorescence spectra, along with the corresponding fluorescence indicator values. The HIX value of AHA aligns with that reported in a previous study [33]. To further evaluate spectral characteristics, we compared the fluorescence peak positions of the HAs with those of the fluorescent dyes used in this study. This comparison revealed that Peak C_1_ was positioned near the fluorescence peak of ANS, making it difficult to assess peak shifts of ANS in the presence of HAs due to spectral overlap. In contrast, the fluorescence peaks of AO, MB, and RhB were well separated from those of the HAs, indicating their suitability for fluorescence-based interaction studies with HAs.

### 3.3. Fluorescence Peak Shifts of AO and MB in the Presence of HAs

AO and MB, which exhibited distinct shifts in their maximum emission wavelengths depending on solvent polarity and whose fluorescence peaks did not overlap with those of the HAs, were selected for further analysis. Specifically, changes in the fluorescence peak positions and intensities of AO and MB were evaluated in the presence of varying concentrations of AHA and WHA and compared to those under HA-free conditions (Table 2). Both dyes exhibited increased fluorescence quenching with rising HA concentrations. Notably, AO displayed a high quenching percentage, making it difficult to clearly detect peak shifts in the presence of HAs. In contrast, MB exhibited a blue shift in its fluorescence peak position with increasing HA concentration. At an HA concentration of 5 mg/L, the WHA sample, which exhibited a higher *E*280 (an indicator of aromaticity) than AHA, was found to show a greater MB peak shift and quenching percentages (Table S1 and Table 2). This shift is likely attributed not only to a decrease in local polarity but also to specific interactions such as electrostatic binding or π–π stacking between MB and the aromatic moieties of HAs. Based on these observations, MB was identified as the most suitable dye among those tested. However, at HA concentrations exceeding 10 mg/L, over 90% quenching of MB fluorescence was observed, which in some cases hindered accurate evaluation of peak shifts. Consequently, an HA concentration of 5 mg/L was adopted as optimal for use with MB in subsequent experiments.

**Figure 4:**
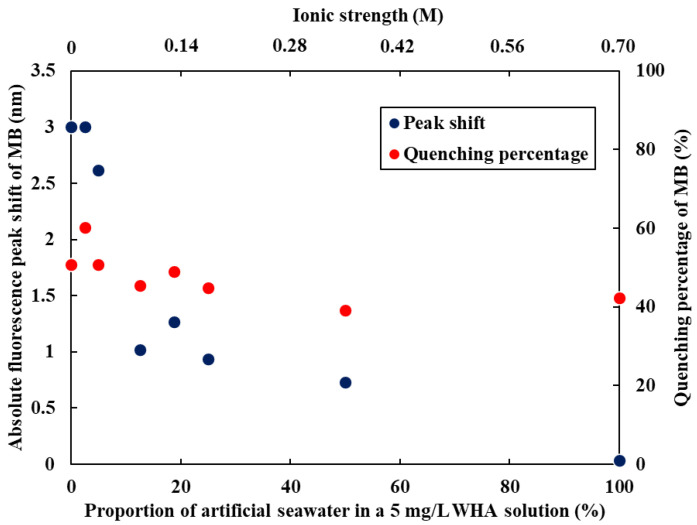
The effect of the proportion of artificial seawater in a 5 mg/L WHA solution on the fluorescence peak shift and quenching percentage of MB.

To explore the applicability of this method to environmental samples, we evaluated the effect of changes in ionic strength on the fluorescence wavelength shift and quenching behavior of MB in the presence of HSs. A series of 5 mg/L WHA solutions with varying proportions of artificial seawater were prepared, and MB was added to each solution. The MB peak shift remained nearly constant (2.6–3.0 nm) when the artificial seawater fraction was up to 5%, corresponding to an ionic strength of 0.02 M (Figure 4). In contrast, at an artificial seawater fraction of 12.5% (ionic strength of 0.04 M), the peak shift decreased to approximately one-third of its original value. Under 100% artificial seawater conditions (ionic strength of 0.7 M), the peak shift was almost completely suppressed. These results are attributed to aggregation of humic acids induced by increased ionic strength, which alters the interaction between MB and HA. The findings indicate that, at present, this method is primarily applicable to the structural characterization of extracted and purified HSs. For direct application to HSs present in real environmental matrices without extraction or purification, it is necessary to further clarify the influence of coexisting components, including ionic strength. On the other hand, the results obtained under artificial seawater conditions suggest that this approach may be useful for comparatively evaluating the aggregation behavior and ionic-strength responsiveness of different HSs.

**Figure 5:**
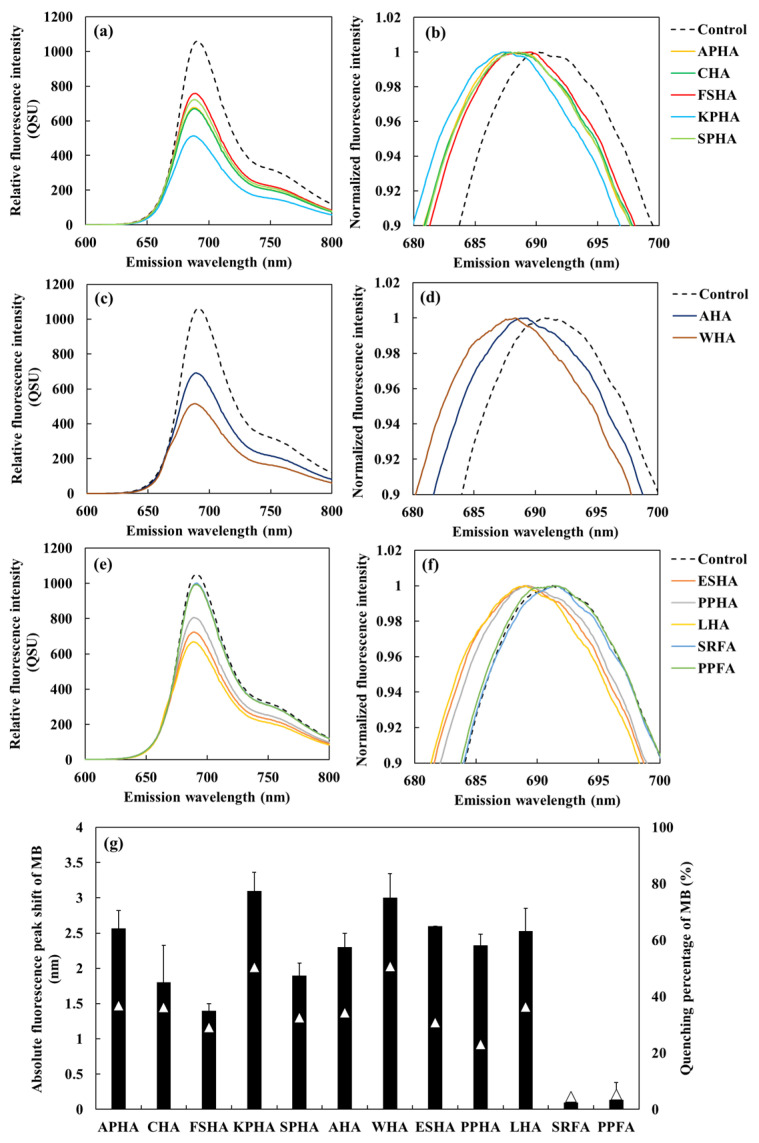
The fluorescence spectrum and normalized spectrum of MB (10µM) under each 5 mg/L humic acids (HAs) and fluvic acids (FAs) extracted from five soil types ((**a**,**b**): APHA, CHA, FSHA, KPHA, SPHA), two commercial sources ((**c**,**d**): AHA and WHA) and IHSS standards ((**e**,**f**): ESHA, PPHA, LHA, SRFA, PPFA). The spectra were processed using Savitzky–Golay smoothing (window size of 31). Normalized fluorescence intensity was calculated by dividing the fluorescence intensity at each wavelength by the peak fluorescence intensity. (**g**) The fluorescence peak shift (black bars) and quenching percentage (white triangles) of MB in the presence of each HA and FA at a concentration of 5 mg/L.

The fluorescence spectrum of MB (10 µM) in the presence of each HA at a concentration of 5 mg/L was recorded in a PB (pH 7.0). The spectra were smoothed using the Savitzky–Golay filter, and the original spectra before smoothing are shown in Figure S2. The results displayed a clear blue shift in the fluorescence peak of MB (Figure 5a–f), indicating that peak shifts can be reliably evaluated at this HA concentration. The corresponding emission wavelength shifts and fluorescence quenching percentages of MB in the presence of various HAs and FAs are summarized in Figure 5g. A strong positive correlation was observed between the absolute peak shift and fluorescence quenching percentages of MB (*r* = 0.910 *p* < 0.001) (Figure S3). A Deming regression accounting for variability in both axes yielded a slope nearly identical to the ordinary least squares fit, confirming the robustness of this relationship.

### 3.4. Fluorescence Peak Shift of MB as an Indicator of HA Characteristics

**Figure 6:**
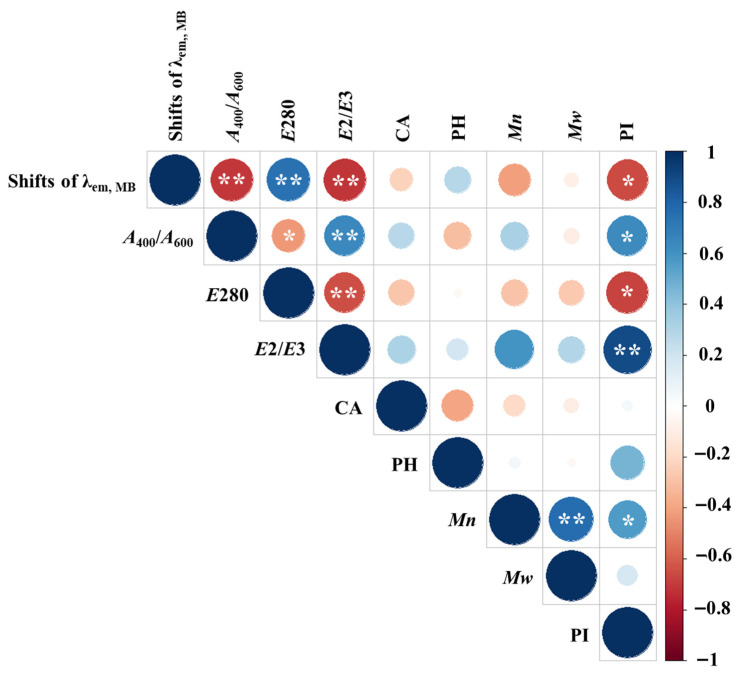
A heatmap of Spearman’s correlation between the absolute fluorescence peak shift of MB in the presence of HAs (Shifts of λ_em, MB_) and various chemical parameters of the HAs and FAs. R-values are represented by colors, while circle diameter indicates the magnitude of the correlation coefficient. A single asterisk (*) denotes a *p*-value < 0.05, and a double asterisk (**) denotes a *p*-value < 0.01. CA: carboxylic acid content; PH: phenolic hydroxyl group content. Samples with no data (ESHA and PPFA) were excluded from the correlation analysis between MB shift and acidic functional groups (CA and PH).

**Figure 7:**
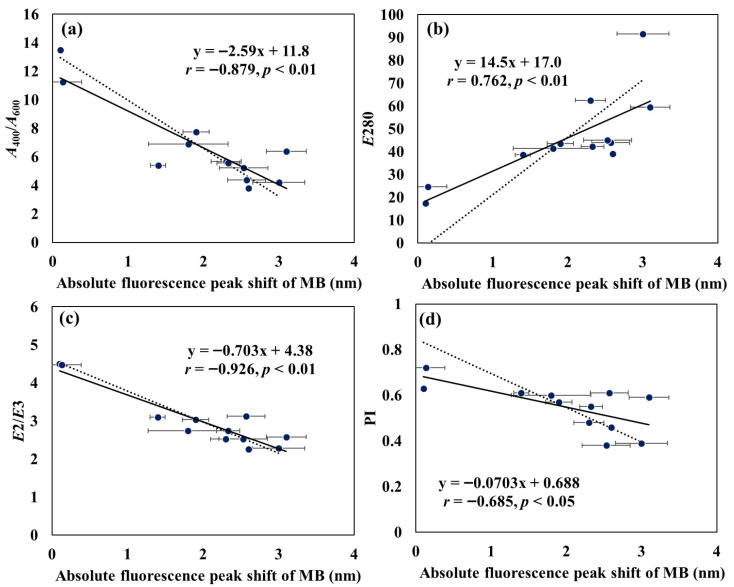
The relationships between the absolute fluorescence peak shift of MB in the presence of different HAs and (**a**) *A*_400_/*A*_600_, (**b**) *E*280, (**c**) *E*2/*E*3, and (**d**) PI The solid lines represent the best-fit ordinary least-squares regression, from which the Pearson correlation coefficients (*r*) and *p*-values shown in each panel were calculated. The dotted lines represent the inverse regression models (x on y) used to account for measurement uncertainty predominantly in the *x*-axis variable. The fitted equations are: (**a**) y = −3.36x + 13.3, (**b**) y = 25.1x − 3.86, (**c**) y = −0.819x + 4.61, (**d**) y = −0.150x + 0.846. The inverse regression analyses were performed in R software using the lm(x~y) model.

Figure 6 displays a heatmap of Spearman’s correlations between the fluorescence peak shift of MB in the presence of HSs and various chemical properties of the HSs. Notably, significant correlations (*p* < 0.05) were observed between the MB peak shift and *A*_400_/*A*_600_, *E*280, *E*2/*E*3, and PI. In contrast, no significant correlations were found with functional group content, *Mn*, and *Mw* (*p* = 0.38–0.98). The MB fluorescence peak shift was positively correlated with *E*280 (*r* = 0.762) and negatively correlated with *A*_400_*/A*_600_, *E*2/*E*3, and PI (*r* = −0.685–−0.926). The relationships between the MB peak shift and *A*_400_/*A*_600_, *E*280, *E*2/*E*3, and PI are shown in Figure 7. To account for the variability in the MB peak shift measurements, inverse regression analyses were performed, and the resulting regression equations were compared with the best-fit ordinary least-squares models. As a result, although minor differences in slope magnitude were observed among the regression models, the direction of the relationships (positive or negative) remained consistent across all parameters. This consistency indicates that the MB peak shift robustly captures variations associated with aromaticity, humification degree, and polarity of HSs, even when accounting for variability in the peak shift measurements.

Solvatochromic shifts are known to be sensitive to multiple microenvironmental factors, including polarity, polarizability, hydrogen-bonding ability, and local rigidity. Although these factors may contribute simultaneously to the spectral response, the observed peak shifts exhibited consistent correlations with several independent chemical descriptors of HA structure. However, contributions from inner-filter effects, dye aggregation arising from electrostatic binding, and changes in microviscosity cannot be completely ruled out. The strong quenching and non-monotonic concentration dependence observed at higher HA concentrations (Table 2) also suggest possible aggregation. While these factors may modulate the spectral response, the consistent correlations still support the utility of MB fluorescence as an empirical probe of HA microenvironmental properties.

It should be noted, however, that this evaluation was conducted using purified HAs under controlled pH conditions, and the applicability of this approach to complex environmental samples has not yet been verified. Future studies will therefore be required to assess the robustness and limitations of MB solvatochromism in heterogeneous environmental matrices. Although recent advances have enabled increasingly detailed molecular-level analyses of HAs, many of these techniques are complex, labor-intensive, and require sophisticated instrumentation and data processing. In contrast, the present method offers a rapid and simple alternative, enabling HS characterization using a single excitation wavelength without extensive sample preparation or multivariate analysis. Taken together, these results indicate that the MB fluorescence peak shift can function as an empirical and facile indicator for assessing the structural and microenvironmental characteristics of HSs, while further validation is required for broader environmental applications.

## 4. Conclusions

This study explored the use of solvatochromic fluorescence for characterizing the chemical properties of HSs. Among the tested dyes, AO and MB exhibited clear solvatochromic responses, and MB in particular showed stable and quantifiable peak shifts at an HA concentration of 5 mg/L. Significant correlations were observed between the MB peak shift and several independent chemical descriptors of HAs, reflecting aromaticity, humification degree, and polarity, indicating that MB solvatochromism can serve as an empirical and facile indicator of HA structural and microenvironmental characteristics.

It should be emphasized, however, that the present correlations were based on purified HAs measured under controlled pH conditions. Although the method successfully differentiates HAs isolated from natural environments, its application to complex environmental matrices—where coexisting constituents such as colloids, inorganic ions, and dissolved organic matter fractions may interfere—has not yet been validated. Therefore, the current approach should be considered semi-quantitative, and further validation using environmentally realistic samples is required to establish its broader applicability.

In summary, while advanced analytical techniques enable detailed molecular-level characterization of HSs, the present approach provides a complementary alternative that is rapid, simple, and requires minimal experimental complexity. By relying on a single excitation wavelength and fluorescence-based detection, this method offers a practical framework for empirical assessment of HA microenvironmental characteristics, particularly for comparative screening purposes. While promising, future studies incorporating diverse environmental matrices and expanded datasets will be essential to fully assess the robustness and environmental relevance of this method.

## Figures and Tables

**Table 1:** Peak position and relative fluorescence intensity (RFI) of Peak C_1_, along with the fluorescence index values (FI, BIX, and HIX) for each HA sample. In the peak position notation, Ex. and Em. represent excitation and emission wavelengths, respectively.

	Peak C_1_		Index		
	Peak Position (Ex./Em. nm)	RFI (QSU)	FI ^1^	BIX ^2^	HIX ^3^
APHA	315/502	10.1	0.61	0.25	13.9
CHA	280/502	7.44	0.71	0.28	14.0
FSHA	280/491	8.90	0.72	0.33	11.6
KPHA	285/496	8.07	0.75	0.23	33.5
SPHA	280/490	6.59	0.83	0.37	12.6
AHA	290/498	21.7	0.76	0.30	59.4
WHA	305/479	39.2	0.87	0.26	155

^1^ FI was calculated as the ratio of emission intensity at 450 nm to that at 500 nm following excitation at 370 nm. ^2^ BIX was determined as the ratio of emission intensities at 380 nm and 430 nm upon excitation at 310 nm. ^3^ HIX was calculated as the ratio of the sum of fluorescence intensities at 435–480 nm to that at 300–345 nm under an excitation wavelength of 254 nm.

**Table 2:** Emission wavelength shifts (λ_em_) and fluorescence quenching percentages of AO and MB in the presence of varying concentrations (0.5–20 mg/L) of AHA and WHA. In the peak position notation, Ex. and Em. represent excitation and emission wavelengths, respectively.

		AO			MB		
	Concentration of HAs (mg/L)	Peak Position (Ex./Em. nm)	Peak Shift of λ_em_ (nm)	Quenching Percentage (%)	Peak Position (Ex./Em. nm)	Peak Shift of λ_em_ (nm)	Quenching Percentage (%)
AHA	0.5	530.0/490	0	61	689.0/665	−1.5	5
	1	530.5/490	+0.5	78	689.5/665	−1.0	13
	5	531.0/490	+1.0	98	687.5/665	−3.0	64
	10	540.0/490	+10	99	687.0/665	−3.5	90
	20	−	−	−	695.0/665	+4.5	97
WHA	0.5	529.5/490	−0.5	46	689.5/665	−1.0	8
	1	530.0/490	0	74	688.0/665	−2.5	20
	5	536.5/490	+6.5	98	686.0/665	−4.5	86
	10	−	−	−	699.5/665	+9.0	97
	20	−	−	−	708.5/665	+18	99

## Data Availability

The data are contained within the article and its Supplementary Materials.

## Supplementary Materials

The following supporting information can be downloaded from https://www.mdpi.com/article/10.3390/s26010107/s1, Table S1: Chemical properties of each humic acid (HA) used in this study; Table S2: Chemical properties of the IHSS standard humic substances (HSs) used in this study; Table S3: The physicochemical properties of the solvents used in this study; Figure S1: Fluorescence spectra of each dye in different solvents; Figure S2: Fluorescence spectrum and normalized spectrum of methylene blue (MB) under each 5 mg/L HSs before smoothing; Figure S3: Relationship between the absolute fluorescence peak shift of MB and quenching percentages in the presence of different HSs. Reference [34] is cited in Supplementary Materials.

## Abbreviations

The following abbreviations are used in this manuscript:

ACTN	Acetone
AHA	Sigma-Aldrich humic acid
ANS	8-Aniline 1-naphtharene sulfonic acid
Ant	Anthracene
AO	Acridine orange
APHA	*Amou* peat humic acid
BIX	Biological index
CHA	Compost humic acid
DMSO	Dimethyl sulfoxide
ESHA	Elliott Soil IV humic acid
EtOH	Ethanol
FA	Fulvic acid
FIX	Fluorescence index
FSHA	Forest soil humic acid
HA	Humic acid
HIX	Humification index
HOPs	Hydrophobic organic pollutants
HS	Humic substance
KPHA	*Kalimantan* peat humic acid
LHA	Leonardite humic acid
MB	Methylene blue
PB	Phosphate buffer
PPFA	Pahokee Peat II fulvic acid
PPHA	Pahokee Peat I humic acid
QSU	Quinine sulfate units
RFI	Relative fluorescence intensity
RhB	Rhodamine B
SPHA	*Shinshinotsu* peat humic acid
SRFA	Suwannee River I fulvic acid
THF	Tetrahydrofuran
3DEEM	Three-dimensional excitation–emission matrix
WHA	Wako humic acid
