# Supplementary material for: Novel Method for Characterizing Humic Substances Using Fluorescent Solvatochromism
Source: Sensors (Basel). 2025 Dec 23;26(1):107. doi: 10.3390/s26010107 (PMC12787976; doi:10.3390/s26010107)
Supplement: Supplementary file 1 [file sensors-26-00107-s001.zip › sensors-4027641-supplementary.pdf]

Supporting Information

Novel Method for Characterizing Humic Substances Using Fluorescent Solvatochromism

Kazuto Sazawa [1,*], Hanae Koyama [1], Yusuke Yamazaki [1], Yoshiki Hara [1], Nozomi Kohama [2], Yustiawati Yustiawati [3], and Hideki Kuramitz [1,*]

[1] Department of Natural and Environmental Sciences, Faculty of Science, University of Toyama, Gofuku, Toyama 930-8555, Japan

[2] Hydrogen Isotope Science Research Center, University of Toyama, 3190 Gofuku, Toyama 930-8555, Japan.

[3] Research Center for Limnology and water resources, National research and innovation agency, Jl. Raya Jakarta-Bogor Km. 46, Cibinong, Bogor 16911, Indonesia.

* Correspondence: sazawa@sci.u-toyama.ac.jp; kuramitz@sci.u-toyama.ac.jp

Contents

1. Experimental procedures for HA characterization: spectroscopic characterization and molecular weight estimation by size exclusion chromatography
2. Chemical properties of the IHSS standard humic substances (HSs) used in this study.
3. The physicochemical properties of the solvents used in this study, including dielectric constant, polarity index, and $E_T(30)$ values.
4. Fluorescence spectra of each dye in different solvents.
5. Fluorescence spectrum and normalized spectrum of methylene blue (MB) under each 5 mg/L HSs before smoothing.
6. Relationship between the absolute fluorescence peak shift of MB and quenching rates in the presence of different HSs.

Experimental procedures for HA characterization

Spectroscopic Characterization

The absorbance values at 250, 280, 365, 400, and 600 nm (A_{250}, A_{280}, A_{365}, A_{400} and A_{600}) were recorded for the calculation of A_{400}/A_{600}, with absorptivity at 280 nm (*E*280) and *E*2/*E*3. *E*280 was calculated as follows: *E*280 (1 cm^{-1} g^{-1} of C) = A_{280}/[HS (g·L^{-1})] × (% C/100).

Molecular weight estimation by size exclusion chromatography

A three hundred μL aliquot of 1 g·L^{-1} HAs solution dissolved in 0.01 M NaOH and 900 μL of 0.01 M phosphate buffer (pH 7.0) and acetonitrile (75/25 = v/v) was mixed. To determine the molecular weight of HAs, a 20 μL aliquot was injected into a Jasco PU-2080 plus Intelligent HPLC pump system equipped with a UV-2075 UV/vis detector (Japan Spectroscopic Co., Tokyo, Japan). The mobile phase consisted of a mixture of 0.01 M phosphate buffer (pH 7.0) and acetonitrile (75/25 = v/v), and the flow rate was set at 0.75 mL·min^{-1}. A TSK-Gel α-M column (Tosoh, 7.8 mm inner diameter × 300 mm) was used as the solid phase, and the column temperature was maintained at 40 °C. The UV absorption of HAs was measured at 260 nm. The reference substances used for the construction of molecular weight calibration curves were sodium salts of polystyrene sulfonates with molecular weights of 1.4 k, 4.3 k, 6.8 k, 13 k, 17 k, 32 k, 49 k, 77 k, 150 k, 350 k, 990 k and 2,600 k Dalton (Fluka, Buchs, Switzerland). The number-average molecular weight (M_n) and weight-average molecular weight (M_w) for each HA were estimated.

Table S1. Chemical properties of each humic acid (HA) used in this study. The data of APHA, FSHA, KPHA, SPHA, and WHA were referenced from Kuramitz et al., 2012 [1]. The data of AHA were from Sazawa et al., 2013 [2]. The data of CHA were from Nanayama et al., 2021 [3]. The partition coefficients of anthracene (log K_{OM} of Ant) and the stability constant of mercury (log K_{ML} of Hg) with HAs obtained by using the fluorescence quenching method [3].

	A_{400}/A_{600} [1]	*E*280 (L/g cm) [2]	*E*2/*E*3 [3]	Total acidity (meq/g)	Carboxyl acid (meq/g)	Phenolic hydroxyl groups (meq/g) [4]
APHA	4.38	43.9	3.12	9.82	4.38	4.36
CHA	6.89	41.4	2.74	5.65	3.06	2.59
FSHA	5.40	38.6	3.10	6.96	3.73	4.74
KPHA	6.38	59.5	2.58	13.13	3.74	9.39
SPHA	7.75	43.5	3.03	5.09	4.02	1.07
AHA	5.67	62.5	2.52	5.64	4.55	1.09
WHA	4.20	91.6	2.28	5.24	2.08	3.16

	M_n (Da) [5]	M_w (Da) [6]	%C	%H	%N	%O	PI [7]	Types [8]	Log K_{OM} of Ant [9]	Log K_{ML} of Hg [9]
APHA	1604	11852	52.2	5.0	3.8	38.3	0.61	P	4.46	5.92
CHA	1474	7592	52.4	5.2	4.7	36.7	0.60	Rp	4.68	5.77
FSHA	2027	14084	51.1	5.2	4.0	37.7	0.61	Rp	4.69	5.89
KPHA	1117	3812	52.9	4.4	1.9	39.6	0.59	B	5.01	5.79
SPHA	1869	18541	52.7	5.1	2.5	37.4	0.57	Rp	4.81	5.77
AHA	891	1548	57.6	4.2	1.0	35.5	0.48	B	5.04	5.62
WHA	490	3081	63.2	3.3	1.3	31.3	0.39	A	5.34	5.34

[1] Ratio of absorbance at 400 and 600 nm [4]. [2] Absorptivity at 280 nm (L/g cm). [3] Ratio of absorbance at 250 and 365 nm. [4] Phenolic hydroxyl group content was calculated by subtracting the carboxylic acid content from the total acidity. [5] Number-average molecular weight was calculated based on sodium polystyrene sulfonate. [6] Weight-average molecular weight was calculated based on sodium polystyrene sulfonate. [7] Polarity Index was calculated from the ratio of O + N to C. [8] Types of HAs classified according to the definition by Ikeya et al. (2003) and Kumada et al. (1967) [4, 5]. The degree of humification of each HA was as follows: A > B > Rp. [9] The partition coefficients of anthracene (log K_{OM} of Ant) and the stability constant of mercury (log K_{ML} of Hg) with HAs obtained by the fluorescence quenching method [3].

Table S2. Chemical properties of the IHSS standard humic acids (ESHA: Elliott Soil IV HA, PPHA: Pahokee Peat I HA, LHA: Leonardite HA) and fulvic acids (SRFA: Suwannee River I FA, PPFA: Pahokee Peat II FA) used in this study.

	**Cat. No.**	A_{400}/A_{600}	***E*280 (L/g cm)**	***E*2/*E*3**	**Carboxyl acid (meq/g)** [1]	**Phenolic hydroxyl groups (meq/g)** [1]	
ESHA	4S102H	3.78	39.0	2.26	No data	No data	
PPHA	1S103H	5.57	42.2	2.74	9.01	1.91	
LHA	1S104H	5.21	45.0	2.53	7.46	2.31	
SRFA	2S101F	13.5	17.3	4.50	11.17	2.84	
PPFA	2S103F	11.2	24.7	4.47	No data	No data	
	***Mn* (Da)**	***Mw* (Da)**	**%C** [1]	**%H** [1]	**%N** [1]	**%O** [1]	**PI**
ESHA	507	6332	59.51	3.20	3.90	32.16	0.46
PPHA	1296	9571	56.37	3.82	3.69	37.34	0.55
LHA	573	5265	63.81	3.70	1.23	31.27	0.38
SRFA	745	3258	52.34	4.36	0.67	42.98	0.63
PPFA	1217	3537	50.45	3.52	2.56	45.47	0.72

[1] Acidic functional groups and elemental compositions were referenced from the IHSS website (https://humic-substances.org/).

Table S3. The physicochemical properties of the solvents (THF, DMSO, ACTN, EtOH, and Water) used in this study, including dielectric constant (ε_r), electric dipole moment of a molecule (μ), refractive index (n_D), and $E_T(30)$ values [6].

Solvent	ε_r	$\mu \cdot 10^{30}$ (Cm)	n_D	$E_T(30)$ (kcal/mol)
THF	7.58	5.8	1.4072	37.4
DMSO	46.45	13.5	1.4793	45.1
ACTN	20.56	9.0	1.3587	42.2
EtOH	24.55	5.5	1.3614	51.9
Water	78.36	6.2	1.3330	63.1

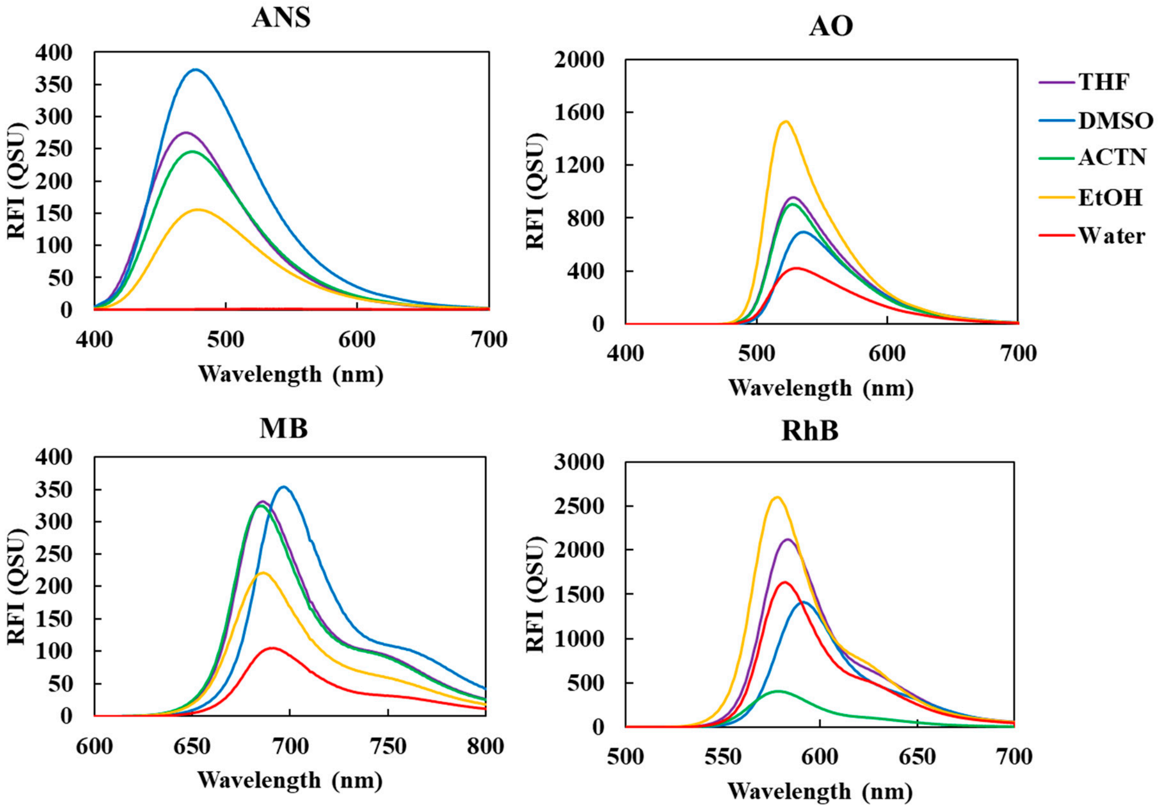

Figure S1 Fluorescence spectra of 10µM each dye (ANS, AO, MB and RhB) in different solvents (THF, DMSO, ACTN, EtOH, and Water).

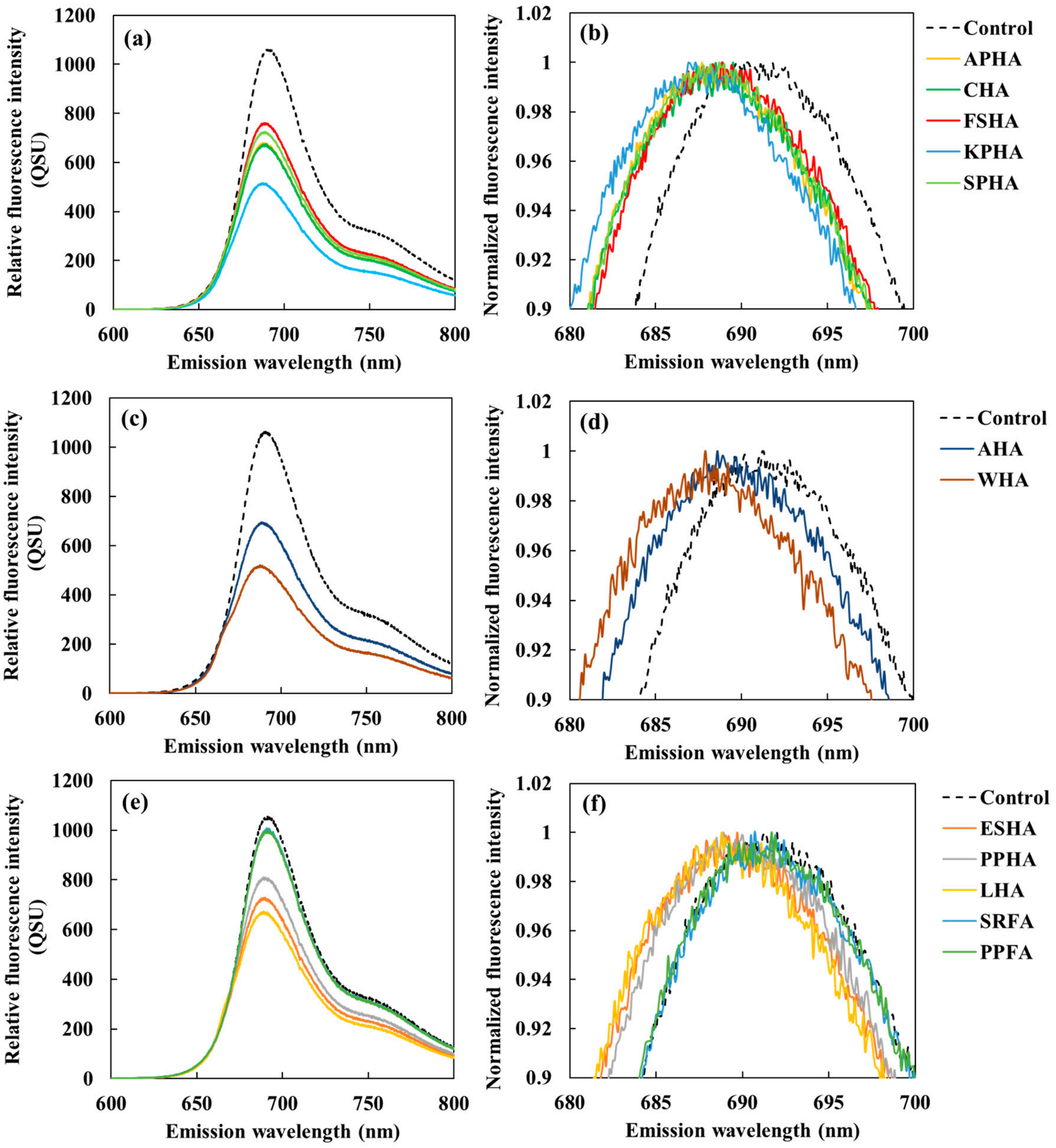

Figure S2 The fluorescence spectrum and normalized spectrum of methylene blue (10μM) under each 5 mg/L humic acids (HAs) and fluvic acids (FAs) extracted from five soil types (a and b: APHA, CHA, FSHA, KPHA, and SPHA), two commercial sources (c and d: AHA and WHA) and IHSS standards (e and f: ESHA, PPHA, LHA, SRFA, and PPFA). The spectra shown here are the original data before Savitzky–Golay smoothing.

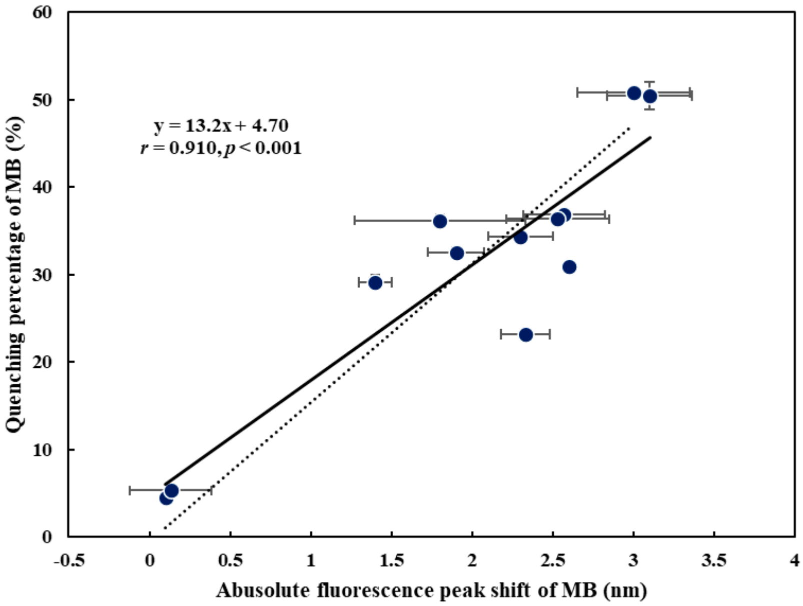

Figure S3 The relationship between the absolute fluorescence peak shift of MB and quenching percentages in the presence of different HSs. The solid line represents the best-fit ordinary least-squares regression, from which the Pearson correlation coefficient (*r*) and *p*-value shown in the panel were calculated. The dotted line represents the Deming regression (errors-in-variables) accounting for uncertainties in both axes, with the fitted equation y = 15.9x − 0.58. The Deming regression was performed using the Deming function in the "MethComp" package of R statistical software.

References

1. Kuramitz, H.; Sazawa, K.; Nanayama, Y.; Hata, N.; Taguchi, S.; Sugawara, K.; Fukushima, M. Electrochemical genotoxicity assay based on a SOS/*umu* test using hydrodynamic voltammetry in a droplet. *Sensors* **2012**, 12, 17414–17432. https://doi.org/10.3390/s121217414
2. Sazawa, K.; Furuhashi, Y.; Hata, N.; Taguchi, S.; Fukushima, M.; Kuramitz, H. Evaluation of the toxicity of tetrabromobisphenol A and some of its oxidation products using a micro-scale algal growth inhibition test. *Toxicol. Environ. Chem.* **2013**, 95, 472–482. https://doi.org/10.1080/02772248.2013.775290
3. Nanayama, Y.; Sazawa K.; Yustiawati, Y.; Syawal, M.S.; Fukushima, M.; Kuramitz, H. Effect of humic acids on the toxicity of pollutants to Chlamydomonas reinhardtii: Investigation by a microscale algal growth inhibition test. *Environ. Sci. Pollut. Res.* **2021**, 28, 211–219. https://doi.org/10.1007/s11356-020-10425-8
4. Ikeya, K.; Watanabe, A. Direct expression of an index for the degree of humification of humic acids using organic carbon concentration. *Soil Sci. Plant. Nutr.* **2003**, 49, 47–53. https://doi.org/10.1080/00380768.2003.10409978
5. Kumada, K.; Sato, O.; Ohsumi, Y.; Ohta, S. Humus composition of mountain soils in Central Japan with special reference to the distribution of P type humic acid. *Soil Sci. Plant. Nutr.* **1967**, 13, 151–158. https://doi.org/10.1080/00380768.1967.10431990
6. Reichardt, D. *Solvents and solvent effects in organic chemistry*, 3rd ed., Wiley-VCH, Weinheim, Germany, 2003.
